# Island biogeography and ecological modeling of the amblypygid *Phrynus marginemaculatus* in the Florida Keys archipelago

**DOI:** 10.1002/ece3.4333

**Published:** 2018-07-30

**Authors:** Kenneth J. Chapin, Daniel E. Winkler, Patrick Wiencek, Ingi Agnarsson

**Affiliations:** ^1^ Department of Ecology & Evolutionary Biology University of California, Los Angeles Los Angeles California; ^2^ Department of Ecology & Evolutionary Biology University of California, Irvine Irvine California; ^3^ Department of Biology University of Vermont Burlington Vermont

**Keywords:** Amblypygi, ecological model, Florida Keys, habitat suitability, island biogeography, MaxEnt, metapopulation, pine rockland, population genetics, urban development

## Abstract

**Aim:**

The biogeography of terrestrial organisms across the Florida Keys archipelago is poorly understood. We used population genetics and spatioecological modeling of the Amblypygi *Phrynus marginemaculatus* to understand the genetic structure and metapopulation dynamics of Keys populations that are otherwise isolated by human development and ocean.

**Location:**

The Florida Keys archipelago and mainland Florida.

**Methods:**

We sequenced a 1,238 bp fragment of mtDNA for 103 individuals of *P. marginemaculatus* from 13 sites in the Florida Keys and South Florida, binned into four regions. We used population genetic analyses to understand the population structure of the species throughout its US range. Furthermore, we used ecological modeling with climate, habitat, and human development data to develop habitat suitability estimates for the species.

**Results:**

We found clear genetic structure between localities. The Lower Keys, in particular, support populations separate from those in other regions studied. Ecological modeling and genetic analyses showed the highest habitat suitability and genetic isolation in the Lower Keys, but urban development across the species range has resulted in the loss of most historical habitat.

**Main conclusions:**

A mainland‐metapopulation model best fits *P. marginemaculatus* gene flow patterns in the Florida Keys and mainland. Ocean currents likely play a role in metapopulation dynamics and gene flow for terrestrial Keys species like *P. marginemaculatus*, and genetic patterns also matched patterns consistent with geologic history. Suitable habitat, however, is limited and under threat of human destruction. The few remaining pockets of the most suitable habitat tend to occur in parks and protected areas. We argue that conservation efforts for this species and others in the terrestrial Florida Keys would benefit from a deeper understanding of the population genetic structure and ecology of the archipelago.

## 1 INTRODUCTION

A central goal of biology is to understand how time and space shaped the evolutionary history of life (MacArthur & Wilson, 1967; Podas, Crisci, & Katinas, 2006; Warren et al., 2015). The Florida Keys is a biodiverse archipelago with high endemism (Forys & Allen, 2005; Kautz & Cox, 2001), but the biogeography of terrestrial populations along the island chain and nearby mainland Florida remains poorly understood. This is particularly surprising considering the Florida Keys was the study site used to develop some of the most iconic biogeography theory (e.g., MacArthur & Wilson, 1967; Simberloff, 1976; Simberloff & Wilson, 1969, Simberloff & Wilson 1970; Wilson & Simberloff, 1969) .

The Florida Keys may support metapopulation spatial structure for some species or genetic divergence and speciation in others (Hanski, 1998; Shrestha, Wirshing, & Harasewych, 2015). In either case, the distribution of species across the Keys is important for understanding both species long‐term survival and biodiversity. This is especially true considering the precariousness of Florida Keys habitats in the face of human disturbance, including human development, deforestation, nonnative species introductions, and human‐induced climate change impacts like sea level rise, increased catastrophic storms, and altered fire regimes (Bancroft, Strong, & Carrington, 1995; Forys, 2005; Maschinski et al., 2011; Ross, O'Brien, & da Silveira Lobo Sternberg, 1994; Ross, O'Brien, Ford, Zhang, & Morkill, 2009). Furthermore, the Florida Keys remain a major tourist destination with over 4.5 million tourists visiting annually (McClenachan, 2013).

Our understanding of the genetic structure of Florida Keys organisms largely comes from research of marine species, where ocean currents play a major role in gene flow and migration (Apodaca, Trexler, Jue, Schrader, & Travis, 2013; DeBiasse, 2010; Kirk, Andras, Harvell, Santos, & Coffroth, 2009; Lacson & Morizot, 1991) . Genetic patterns of terrestrial species are expected to differ considerably, as the marine ecosystem acts as an uninhabitable matrix and the formation of terrestrial habitat occurred on different timescales (Hoffmeister & Multer, 1968; Shrestha et al., 2015).

Genetic research using native terrestrial species is scarce, but not absent. The mosquito *Aedes aegypti* showed no genetic structure along the Florida Keys, likely because they disperse via flight (Brown, Obas, Morley, & Powell, 2013). The invasive brown anole (*Anolis sagrei*) and greenhouse frog (*Eleutherodactylus planirostris*) showed introductions to the Florida Keys from Cuba but cannot inform patterns for native species across the Keys (Heinicke, Diaz, & Hedges, 2011; Kolbe et al., 2004). An allozyme electrophoretic study on the Florida Tree Snail (*Liguus fasciatus*) revealed low levels of genetic diversity, likely due to a recent introduction from Cuba, or the low resolution of allozyme approaches (Hillis, Dixon, & Jones, 1991). The land snail *Cerion incanum* showed some haplotype structure between Upper and Lower Keys, likely caused by differences in the timing of formation of the Keys. Shrestha et al. (2015) proposed that the *C. incanum* spread southwesterly to colonize new Keys as they formed, with Lower Key populations being the youngest. Lastly, ant gut microbiota showed genetic structure between the upper and lower keys (Hu et al. 2013). That said most past biogeographic studies have been limited to nonnative species or excluded Florida mainland populations. No studies have investigated the biogeography of a native species occupying the entire archipelago and mainland, or have any investigated human impacts on structure and connectivity.

The human population of the Florida Keys has drastically impacted ecosystems therein. The human population of Monroe County, which includes the Florida Keys and a portion of rural land west of Everglades National Park, has more than doubled since 1950 (US Census Bureau). While population sizes of residents may have stabilized over the last 25 years (currently ca. 77,000 residents), the number of tourists visiting the Keys is enormous. In 2014, an estimated 4.516 million tourists visited the Florida Keys (Key West Chamber of Commerce, 2017). Key West, the major city of the Florida Keys, located on an island of only 19 km^2^, has over 52,000 housing units. The natural areas that remain are mostly protected as state parks or national wildlife refuges, but are continually impacted by nearby human activity (Peterson, Lopez, Frank, Porter, & Silvy, 2004).

We used field observations coupled with land use and climate data to model *Phrynus marginemaculatus* distribution in South Florida and the Florida Keys. Species distribution models (SDM) have been employed in many conservation, evolutionary, and ecological applications (Elith & Leathwick, 2009). These include studies of spatial patterns of diversity (Hoagstrom, Ung, & Taylor, 2014; Peterson, 2011; Waltari & Guralnick, 2009) , genetic structure (Gotelli & Stanton‐Geddes, 2015), and the historic spread of invasive species (Li, Dlugosch, & Enquist, 2015; Václavík, Kupfer, & Meentemeyer, 2012) . Additionally, SDM have identified suitable habitat for species of concern (Tittensor et al., 2009) and identified climatic factors driving species distributions, including responses to climate change (Feng & Papeş, 2015; Ficetola, Thuiller, & Miaud, 2007). Additionally, SDM techniques have advanced in the past decade (Guisan & Thuiller, 2005) to allow for predictive power with presence‐only data (Bradley, 2015; Bradie & Leung, 2016; Elith et al., 2006; Jiménez‐Valverde, Decae, & Arnedo, 2011) and small samples (Pearson, Raxworthy, Nakamura, & Peterson, 2007; Proosdij, Sosef, Wieringa, & Raes, 2016; Wisz et al., 2008).

We aimed to quantify the genetic and ecological characteristics of *P. marginemaculatus* populations in the US. In particular, we aimed to uncover the genetic structure of the species across the Florida Keys archipelago, understand the evolutionary history of Keys populations in relation to the species range via phylogenetic analysis, and identify suitable habitat and locations of putative populations throughout the species’ potential range. Together, these results will be the first to examine *P. marginemaculatus* in the wild, and will provide critical information applicable to many terrestrial island species and their conservation.

## 2 MATERIALS AND METHODS

### 2.1 Study site

The Florida Keys is a ca 250‐km‐long archipelago amounting to ca 350 km^2^ of dry land, extending from Key Largo bordering mainland Florida southwest to Key West 140 km from Cuba. The Keys are made from two geologic formations that both formed during the Tarantian Pleistocene (0.126–0.0117 mya) and raising above sea level during the Wisconsin glaciation (ca. 100,000 before present; Hoffmeister & Multer, 1968; Shrestha et al., 2015). The Lower Keys (Big Pine Key to Key West) formed from cemented sand bars, resulting in oölitic limestone (termed Miami Limestone). These Keys curve west and orient laterally due to gulf stream currents. The Upper Keys (Bahia Honda Key to Key Largo), however, are constituted of fossil coral reefs (termed Key Largo Limestone) without strong lateralization. Some species show endemism to the Lower or Upper Keys, but not both because of these differences (e.g., Peck & Howden, 1985).

Florida mixed hardwood forests have persisted since the birth of terrestrial Florida 25 mya (Webb, 1990). Since then, species have had two routes to colonizing Florida habitats: a land migration down the Florida peninsula from North America, or water migration north or west from the Caribbean and Bahamas, as Florida was never connected to the Caribbean islands (Snyder, 1990). Thus, we might expect the diversity of Florida to be shaped by tropical species able to disperse over water, and temperate species only able to establish via land. This has resulted in a dominance of vertebrates from North America but flora from the Caribbean (Snyder, 1990). Exceptions include nine bird species, and two species each of bat, frog and lizard, all of which have Caribbean origins. That being said, natural migrations are only clear for a few of these species; many might have been introduced via humans, and still others have gone extinct (at least locally) since their discovery (Snyder, 1990). Invertebrate biogeographic patterns are more mixed, but still fit the model of tropical water migrators versus temperate land migrators. For example, most Florida ant species have North American origins, while Butterflies are largely Caribbean (Lenczewski, 1980).

Land to support the growing human populations of Miami and the Florida Keys have been largely obtained by clearing pineland and hammock (Snyder, Herndon, & Robertson, 1990), a major conservation issue that researchers have been bringing attention to for nearly a century (Small, 1929). The first settlers in Southern Florida were concentrated in the Florida Keys. Early settlers exploited pine and hardwood trees (especially Mahogany) for lumber, fuel, and slash‐and‐burn agricultural practices (Small, 1917; Browder, Littlejohn, & Young, 1976; Wilson & Porras, 1983) . As a result, very few stands of rockland include original forest. Industrial logging was enabled by the Florida East Coast Railroad, which reached Miami in 1896. Rockland habitat was subject to clear‐cutting for timber and fuel but made poor agricultural land due to an abundance of limestone rocks that made soil unworkable. Greatly expanding agriculture oftentimes spared rockland habitat in favor of draining glades for crops until the invention of the rock plow in the 1950s. This enabled limestone rocks to be collected and separated from soil, thereby enabling agricultural access. Limestone rocks collected via hand, plow, and mine, were used as building materials and can be seen in historical buildings, walls, and gardens of the Florida Keys today. Rocklands are nutrient and water depauperate, and thus require the heavy use of irrigation and chemical fertilizers to be agriculturally usable. As a result, abandoned rocklands show little resemblance to the original ecosystem, and are often dominated by invasive species (Loope & Dunevitz, 1981). The ability to turn rockland into space for agriculture and housing has led to the steep decline in rockland habitats that continue to the present, with practically no hope of reestablishment without human intervention (Dorn, 1956; Meyers & Ewel, 1990; Possley, Maschinski, Maguire, & Guerra, 2014; Snyder, 1990).

### 2.2 Study species

**Figure 1:**
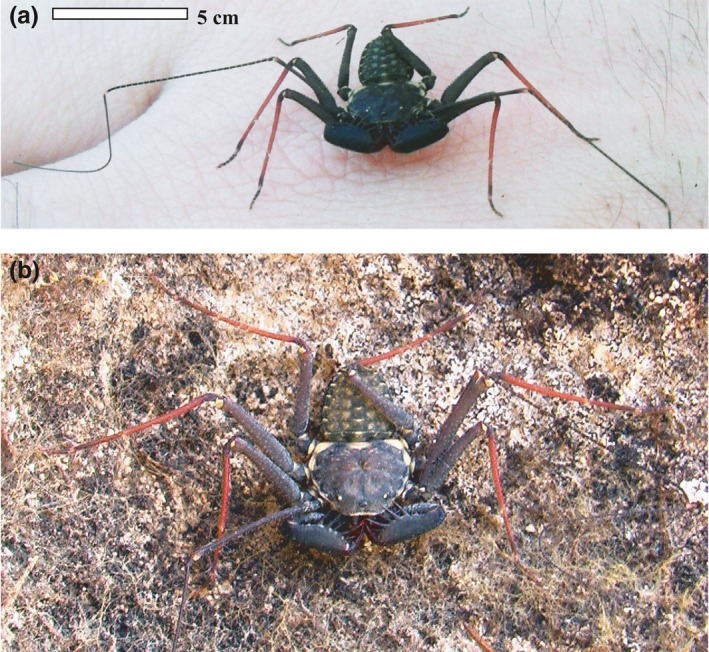
Photographs of the amblypygid *Phrynus marginemaculatus* in the Florida Keys. (a) Note the elongated first pair of legs adapted as sensory structures. (b) Close up of the body highlights coloration and patterning

We used the amblypygid species *P. marginemaculatus* C.L. Koch, 1840 as a model to understand the general biogeographic pattern of terrestrial Florida Keys species. Amblypygids are a small arachnid order (ca. 220 spp.) of large nocturnal predators (Chapin & Hebets, 2016). *Phrynus marginemaculatus* is the only amblypygid species in the US east of the Mississippi river and the most commonly studied species of amblypygid (Chapin & Hebets, 2016; Figure 1). Laboratory research has shown that *P. marginemaculatus* exhibit ritualized agonistic displays (Fowler‐Finn & Hebets, 2006), and can learn to navigate mazes using tactile cues (Santer & Hebets, 2009a, 2009b) via exceptional brain structures and sensory systems (Chapin & Hebets, 2016; Santer & Hebets, 2011). While fascinating laboratory research has been conducted on the species, no research on their habitat requirements, distribution, population ecology, or population genetics has ever been published (Chapin & Hebets, 2016; Weygoldt, 2000).

Historical records indicate that the species was found as far north as Martin County, FL, but recent records are absent. The species is also found on several Bahamian islands, Cuba, Jamaica, and Hispaniola (Muma, 1967; Quintero, 1981). Research on the species, however, has only occurred in captivity, with animals collected from a single island (Big Pine Key; Hebets & Chapman, 2000; Fowler‐Finn & Hebets, 2006; Spence & Hebets 2006; Santer & Hebets, 2009a, 2009b) , the pet trade (Rayor & Taylor, 2006), or both (Graving, 2015).

Interestingly, *P. marginemaculatus* has evolved a plastron to breathe underwater, which they can do for upwards of 24 hr (Hebets & Chapman, 2000). While the function of the plastron is not well understood, it likely increases chances of survival during flooding in their terrestrial retreats. This may be particularly important in the Florida Keys, where annual hurricanes can result in flooding. Furthermore, hurricanes, along with ocean currents, likely promote oceanic dispersal (Fleming & Murray, 2009; Gillespie et al., 2011) . A plastron allowing underwater breathing likely extends the dispersal propensities across bodies of water, and the likelihood of survival during ocean migration, in *P. marginemaculatus*.

Two molecular phylogenetic studies have focused on amblypygids. First, a phylogeny of the *Damon variergatus* group delineated two cryptic species within *Damon*, an African genus of amblypygid (Prendini, Weygoldt, & Wheeler, 2005). Second, phylogenetic analyses of *Phrynus* species in Puerto Rico revealed hidden dimensions of diversity across cave populations (Esposito et al., 2015). In particular, Esposito et al. (2015) noted high levels of diversity across mitochondrial but not nuclear, genomes. Here we focus on mitochondrial sequences to examine if similar genetic structure across small geographical scales is evident in the Florida Keys.

### 2.3 Specimen collection

**Figure 2:**
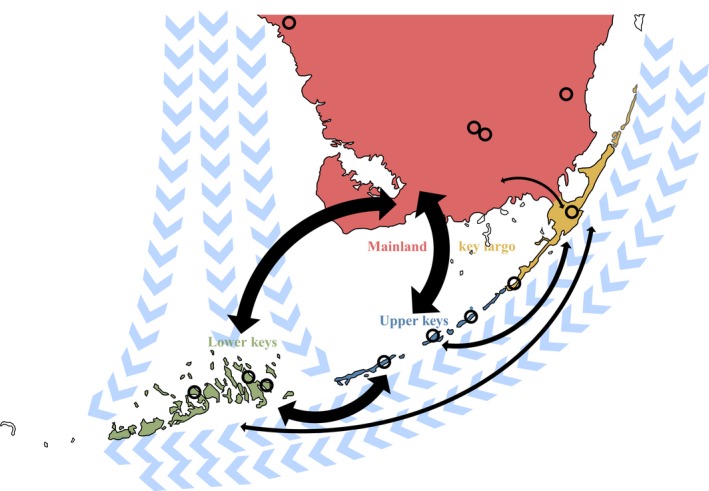
Map of Southern Florida with geographic regions (colors), ocean currents (blue arrows; adapted from Lee & Smith, 2002), localities where *Phrynus marginemaculatus* samples were collected (open circles), and pairwise *G*_ST_ (thicker lines indicate lower *G*_ST_ t; *G*_st_ range: 0.174–0.620) indicating migration

We collected *P. marginemaculatus* specimens from 13 locations in southern mainland Florida and the Florida Keys archipelago using a nonrandom sampling method (Table 1; Figure 2). We limited our survey to upland habitat types, as we assumed that *P. marginemaculatus* would not be found in intertidal habitats like mangrove swamps and floodplains. Additional survey areas were selected from historic records, which were all associated with these upland habitat types (Table 1). *P. marginemaculatus* hide under debris, especially limestone rocks, during the day (Chapin & Hebets, 2016; Hebets & Chapman, 2000). Our sampling regime included walking trails and looking for *P. marginemaculatus* under rocks, logs, and other larger debris. When found, we collected genetic samples and stored them in 95% ethanol on dry ice. We dissected muscle tissue from one or a few appendages, depending on the size of the specimen.

**Table 1:** Genetic diversity indices of COI sequences for major regions in which *Phrynus marginemaculatus* occur

pop	*N*	*H*	*G*	*λ*	*λc*	*H*_exp_	*π*
Ley Largo	18	2.44	8.53	0.883	0.935	0.032	0.003
Mainland	28	3.12	20.63	0.952	0.987	0.105	0.010
Upper Keys	13	2.56	13.00	0.923	1.000	0.150	0.013
Lower Keys	44	3.54	28.47	0.965	0.987	0.115	0.010
Total	103	4.39	67.57	0.985	0.995	0.131	0.013

*Note*

*G* is Stoddart and Taylor's index of MLG diversity; *H* is the Shannon–Wiener Index of multilocus genotype (MLG) diversity; *H*_exp_ is Nei's unbiased gene diversity; *π* is nucleotide diversity; *N* is the number of individuals sequenced; *λ* is Simpson's Index; *λc* is Simpson's index corrected for variation in sample size.

### 2.4 Extraction, amplification, and sequencing

We extracted genomic DNA using QIAGEN DNeasy Blood & Tissue Kits. We followed the standard kit protocol but used chilled ethanol (−20°C) and a 50‐mL final elution. We amplified a 1,238 nucleotide sequence of the mitochondrial gene cytochrome *c* oxidase subunit 1 (COI) by performing 34 iterations of the following cycle on a thermal cycler: 30 s at 94°C, 35 s at 48°C, and 90 s at 65°C, beginning with an initial cycle of 2 min at 94°C and ending with 10 min at 72°C. Using illustra PuReTaq Ready‐To‐Go PCR beads and 400‐nM forward and reverse primers, the long or short read of COI was sequenced for each sample. LCOI1490 was used as the forward primer for both the long and short reads, while HCOI2198 was used as the reverse primer for the short reads and C1‐N‐2776 was used as the reverse primer for the long reads (LCOI1490 GGTCAACAAATCATAAAGATATTGG, HCOI2198 TAAACTTCAGGGTGACCAAAAAATCA, and C1‐N‐2776 GGATAATCAGAATATCGTCGAGG; Folmer, Black, Hoeh, Lutz, & Vrijenhoek, 1994). We chose a mtDNA sequence as mtDNA is much more informative than nuclear DNA among Amblypygi (Esposito et al., 2015).

### 2.5 Cleanup and alignment

Amplified fragments were sent to the University of Arizona Genetic Core and Genewiz for sequencing. Subsequently sequences were assembled using the Chromaseq module (Maddison & Maddison, 2016a) in Mesquite 3.02 (Maddison & Maddison, 2016b) through Phred and Phrap (Ewing & Green, 1998; Ewing, Hillier, Wendl, & Green, 1998; Green, 1999; Green & Ewing, 2002), and then proofread in Mesquite. Sequences were aligned with ClustalW2 (Larkin et al., 2007) in Mesquite.

### 2.6 Genetic analysis

We grouped localities into four regions by major geologic features: the mainland, Key Largo, Upper Keys (excluding Key Largo), and Lower Keys. We produced genetic diversity indices for each region and used a hierarchical analysis of molecular variance (AMOVA; Excoffier, Smouse, & Quattro, 1992) to estimate the variance within and between localities and regions. We tested for isolation by distance (IBD) by testing for a correlation between Nei's genetic distance and maximum geographic distance among samples with a mantel test (Mantel, 1967). We used discriminant analysis of principal components (DAPC) with cross‐validation to examine genetic divergence between regions and calculated pairwise *G*_ST_ as an estimate of migration between regions. We used the R 2.3.2 (R Core Team) packages “ade4” (Dray & Dufour, 2007), “adegenet” (Jombart, 2008; Jombart & Ahmed, 2011), “mmod” (Winter, 2012), “pegas” (Paradis, 2010), and “poppr” (Kamvar, Brooks, & Grünwald, 2015; Kamvar, Tabima, & Grünwald, 2014) for genetic analyses.

### 2.7 Ecological modeling

We used locality data from the 103 field‐collected specimens to estimate the geographic range of *P. marginemaculatus* using the niche modeling software Maxent 3.3.3 (Phillips, Anderson, & Schapire, 2006; Phillips & Dudík, 2008). Maxent uses a maximum‐entropy algorithm to predict species geographic ranges using presence‐only data and environmental GIS layers. We evaluated 19 BioClim climate variables (BIO1–19; Hijmans, Cameron, Parra, Jones, & Jarvis, 2005) at a 30‐arc‐second resolution (ca. 1 km^2^) for inclusion in our models. We included elevation and a geologic map in our first set of models to test their contribution to informing model predictions. Both layers were obtained from the United States Geological Survey (http://www.usgs.gov). We ran a second set of models that included land use data based on imagery made publically available by the Florida Department of Environmental Protection (http://geodata.dep.state.fl.us/). This dataset included 195 categories of land use based primarily on human use (e.g., agriculture, urban development, transportation corridors) but also included subcategories of vegetation and other ecologically relevant habitat types (e.g., pinelands, mangrove swamps, cabbage palm hammock). This included all land cover categories used in the Florida Land Cover Classification Systems (Kawula, 2009).

All data were clipped to a regional extent of southern Florida and the Florida Keys at approximately latitude 28°N using ArcGIS v10.2.2. This northern latitude is located approximately along the freeze line in Florida (Miller & Glantz, 1988). There is no evidence to indicate that *P. marginemaculatus* occurs beyond this line (Quintero, 1981). We tested all layers for pairwise correlation across the study area using the package ‘Raster’ in R 3.3.2 (Hijmans & van Etten, 2012). We retained 12 of the 19 BioClim layers that had correlation coefficients under |0.75|. These climate variables represent annual and seasonal trends, as well as extremes in temperature and precipitation. Temperature variables included annual mean temperature, mean diurnal range, isothermality, and mean temperatures of both the wettest and driest quarters. Precipitation variables included annual temperature, precipitation during the wettest and driest months, precipitation seasonality, and precipitation of the warmest and coldest quarters.

We ran 100 model replicates using a randomly selected 75% of the occurrence records to calibrate the model and 25% to test it (Phillips et al., 2006), well beyond the ideal minimum sample size to obtain reliable results (Proosdij et al., 2016). Each model was assessed with the area under the receiver operating characteristic curve (AUC; Hanley & McNeil, 1982). AUC values represent a measure of the MaxEnt model's ability to discriminate between suitable and unsuitable areas in the modeled distribution (Anderson & Gonzalez, 2011). AUC values range from zero to one, with one indicating a perfect differentiation of suitable and unsuitable habitat. We compared predicted models against distribution literature for *P. marginemaculatus* (Quintero, 1981). Models that performed poorly (AUC scores < 0.75) or that varied substantially from historical records were discarded. Jackknife tests were used to evaluate the importance of each environmental and abiotic variable to explain the range of *P. marginemaculatus*. Last, we calculated areas of overlap between human land use features (i.e., urban and rural developments, transportation, communication, and utilities, and agricultural lands) and model outputs of suitable habitat using the image‐processing software ImageJ (Abramoff, Magalhaes, & Ram, 2004). We calculated declines in suitable habitat at four thresholds of modeled suitable habitat (>0.1, 0.1, 0.3, 0.5, and 0.9) caused by human development.

## 3 RESULTS

### 3.1 Population genetics

**Figure 3:**
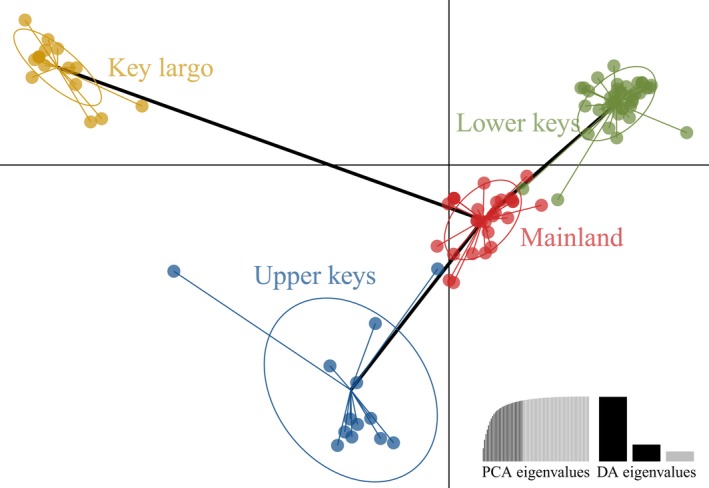
Discriminant analysis of principal components (DAPC) of *Phrynus marginemaculatus* population regions PCA and DA Eigen values is presented as insets. Dashed line is the minimum spanning tree of regions. Stratified cross‐validation of DAPC resulted in a mean successful assignment of 0.75% and 93.9% conserved variance with 10 principal components and three discriminant functions. All regions separated into distinct clusters

We sequenced a 1,238 bp region of the mitochondrial COI gene of 103 individuals. Sequences had an overall base composition of 24.6% adenosine, 25.2% cytosine, 16.5% guanine, and 33.7% thymine. Key Largo exhibited the lowest genetic diversity among regions, with the Lower Keys and mainland regions exhibiting considerably higher genetic diversity (Table 1). Additionally, a range‐wide mantel test for IBD of geographic coordinates and Nei's distance was nonsignificant (Mantel *r *= −0.07, *P *=* *0.637). A hierarchical AMOVA revealed population structure among populations, but not regions (Table 2). The AMOVA indicated significant genetic structure between localities, suggesting that oceans limit dispersal. Stratified cross‐validation of DAPC resulted in a mean successful assignment of 0.92% and 88.2% conserved variance with 20 principal components. All regions separated into distinct clusters, further evidencing regional genetic structure (Figure 3). Furthermore, pairwise *G*_ST_ showed relatively high divergence of Key Largo localities from the rest of the range, and low divergence between mainland and island sites, indicating ongoing gene flow (Figure 2).

**Table 2:** Hierarchical analysis of molecular variance (AMOVA) using cytochrome *c* oxidase I (COI) sequences of *Phrynus marginemaculatus* in Florida indicating genetic structure

	*σ*	% variance	*ϕ*	*p*
Within localities	1.377	38.37	0.616	<0.001
Between localities	1.997	55.65	0.592	<0.001
Between regions	0.214	5.97	0.060	0.160

### 3.2 Ecological modeling

**Figure 4:**
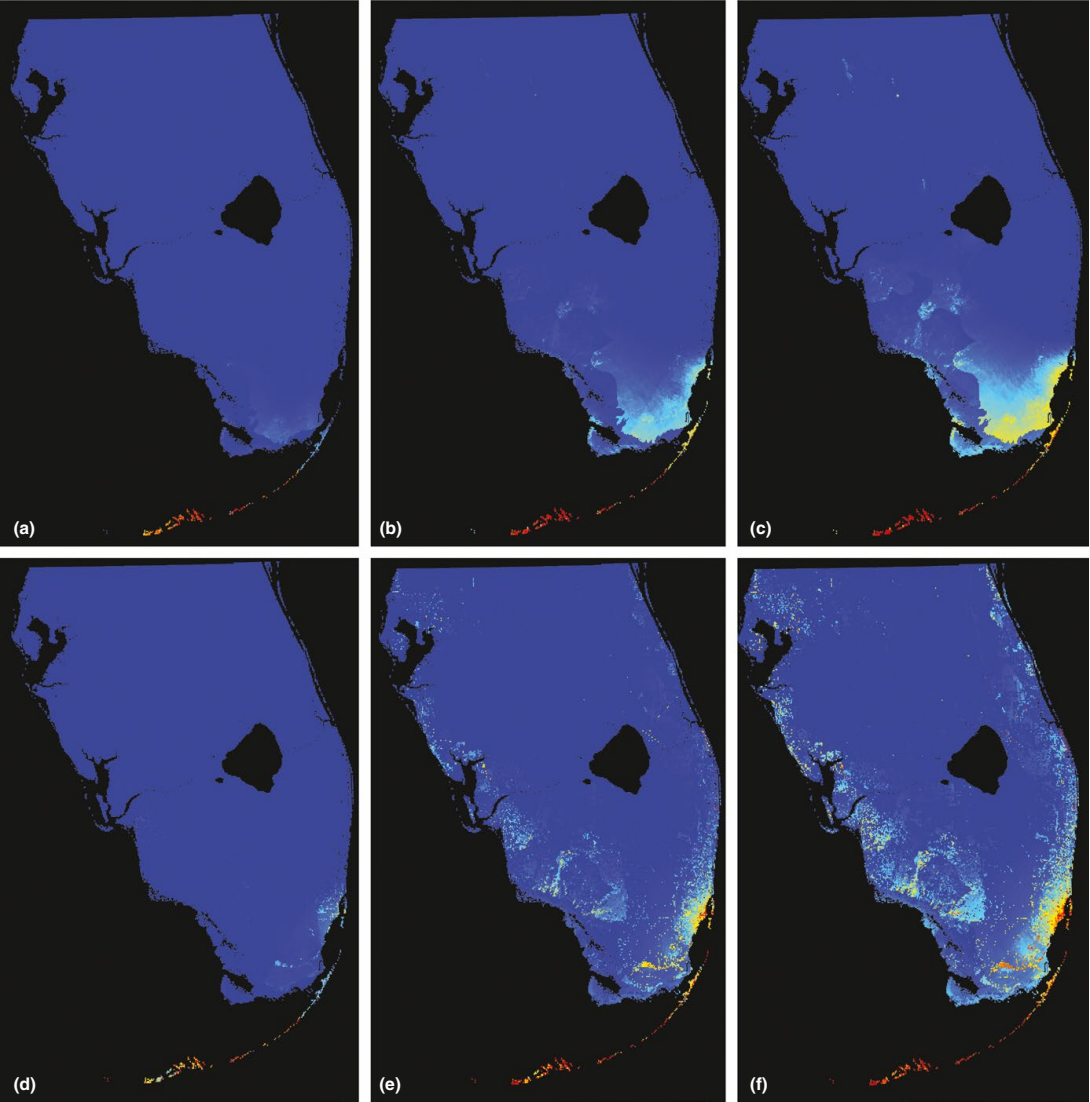
MaxEnt suitability map for *P. marginemaculatus* in southern Florida. Color scale indicates probability of occurrence based on presence‐only data. Minimum, mean, and maximum suitabilities using only climate datasets (a–c; mean AUC = 0.92 ± 0.02); minimum, mean, and maximum suitabilities using climate and vegetation communities datasets (d–f; mean AUC = 0.87 ± 0.6)

Species distribution modeling using MaxEnt found good model fit for climate‐only models (mean AUC = 0.978 ± 0.02, *n *=* *100 models; Figure 4a–c). Habitat suitability was highest in the Florida Keys (Figure 4), but also extended to the southeastern end of mainland Florida. Areas of predicted suitable habitat on the southeastern mainland corresponded to the geologic features of the peninsula, which had a permutation importance of 5.8%. However, suitable habitat was identified primarily by environmental variables that contributed most to the model: precipitation of the coldest quarter (74.8% permutation importance) and mean diurnal temperature range (14.7%). Altitude also had predictive power with 2.4% permutation importance in the first set of models. Jackknife tests of variables in isolation from all others revealed that annual mean temperature had the highest training gain for models, followed by mean temperature of the driest quarter, mean diurnal temperature range, and mean temperature of warmest quarter.

Models including land use categories performed slightly worse (mean AUC = 0.873 ± 0.06, *n* = 100 models) than those without land use but appear to have refined the habitat suitability of *P. marginemaculatus* (Figure 4d–f). Land use categories had the highest permutation importance (50.5%) followed by mean temperature of the driest quarter (18%), mean diurnal temperature range (12.7%), geology (7%), and altitude (3%). Similar to our models without land use categories, annual mean temperature had the highest training gain for models but varied in subsequent variable importance. Mean temperature during the driest quarter, mean diurnal temperature range, and mean temperature of the warmest quarter followed in terms of variable importance in isolation. Models with land use identified regions of the Lower and Upper Keys, pockets in Everglades National Park, and several coastal areas of Miami as the most suitable habitat. Additional suitable areas were identified on Key Largo and in Big Cypress National Preserve. Smaller pockets of potential habitat ranged up the east and west coasts.

**Figure 5:**
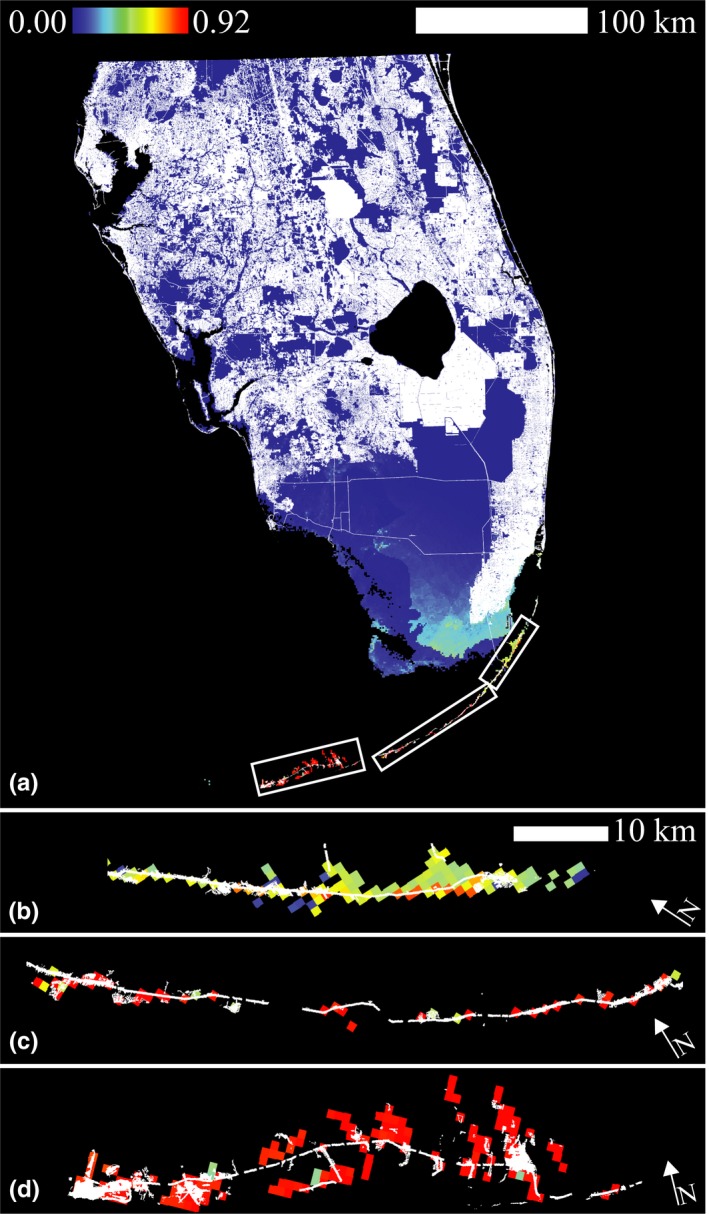
(a) MaxEnt suitability map of the best fit model (climate‐only mean) with human development overlay for the amblypygid *Phrynus marginemaculatus* in the Florida Keys and South Florida. White areas indicate land converted for human use. Panels b–d are 10× magnification of areas indicated in panel a, which include (b) Key Largo, (c) the other Upper Keys, and (d) the Lower Keys. Arrows indicate north. The Keys include particularly high suitability habitat, especially the Lower Keys.46

We saw an alarming 22%–34% human‐induced decline in suitable habitat under the best fit model (Figure 5; Tables 3 and 4). Models that included land use showed steeper declines of 29%–48% of suitable habitat due to human development (Tables 3 and 4). This indicates that, while climatically identified habitat shows considerable decline, human impacts particularly target land use habitat types important for the species.

**Table 3:** Area of suitable habitat of the amblypygid *Phrynus marginemaculatus* in southern Florida and the Florida Keys. Area of habitat with suitability thresholds of 0.1, 0.5, and 0.9 under a climate‐only and climate‐plus land use MaxEnt models. Loss indicates percent loss of habitat at a given threshold by human development. Model fit is indicated by the Area Under the Curve and standard deviation (AUC ± *SD*)

Model	0.1 threshold		0.5 threshold		0.9 threshold		AUC ± *SD*
	km^2^	% loss	km^2^	% loss	km^2^	% loss	
Climate only	3,545.88	27.58	179.95	34.91	4.64	22.44	0.978 ± 0.02
Climate + land use	2,427.96	48.68	300.97	43.81	12.74	28.65	0.873 ± 0.60

**Table 4:** Reduction in suitable habitat for the amblypygid *Phrynus marginemaculatus* predicted by MaxEnt modeling caused by human development in South Florida and the Florida Keys. Threshold is the lower limit for the index of habitat suitability; Habitat is the total area in km^2^ without human development

Threshold	Habitat	Development^a^	Percent loss
Climate‐only (AUC = 0.98 ± 0.02)			
0.1	3,545.88	2,567.79	27.58
0.3	532.32	400.15	24.83
0.5	179.95	117.12	34.91
0.9	4.64	3.60	22.44
Climate + Land use (AUC = 0.873 ± 0.6)			
0.1	4,247.96	2,179.85	48.68
0.3	958.57	558.18	41.77
0.5	300.97	169.11	43.81
0.9	12.74	9.09	28.65

a Development is the reduced area after considering habitat degraded by human use.

## 4 DISCUSSION

Pine rocklands succeed to tropical hardwood hammocks after two or three decades of fire suppression (Loope & Dunevitz, 1981; Robertson, 1953), but it remains unclear if *P. marginemaculatus* occurs in both habitats. Both periodic seawater flooding and fire occur naturally in rockland habitats (Snyder et al., 1990) and *P. marginemaculatus*, like much of the fauna and flora species in these habitats (Hofstetter, 1975; Robertson, 1953, 1962), have evolved to survive these stochastic events.

The mainland and Lower Keys show the greatest genetic diversity. This is likely because these two regions include the largest expanses of area, and both are represented by small patches of suitable habitat within an inhospitable matrix. Key Largo showed relatively low diversity, despite the island's size. However, only a small portion of Key Largo's land area is suitable habitat—most of the island is highly developed.

Genetic variation was significant between localities but not larger regions (Table 2), suggesting a more complicated genetic structure than our a priori regional delineations. Population‐level genetic structure is aligned with intuition, considering all populations in our study occur on Keys or islands of habitat surrounded by human disturbance (Figure 2). Regional structure, however, is somewhat surprising. We selected regions a priori based on geologic features: the Key Largo region for its size and proximity to the mainland; the Upper and Lower Keys for their geologic variation in formation, if not timing thereof; and the mainland, as an obvious delineator from island populations. This generally aligns with the population structure of native *Cerion* land snails, which showed isolation between the Upper and Lower Keys (Shrestha et al., 2015). Species on the Lower Keys likely have a unique evolutionary history separate from the rest of the Keys.

Our DAPC analysis shows clear genetic structure across regions, with key populations all being closest related to mainland localities. This pattern suggests that a mainland–metapopulation model described the landscape genetics of the Keys. As mentioned, genetic analyses of land snails in the Florida Keys also show an Upper–Lower Key division (Shrestha et al., 2015). But research on marine species like bicolor damselfish (*Eupomacentrus partitus*) and common reef sponge (*Callyspongia vaginalis*) found much lower levels of divergence between regions than we found for *P. marginemaculatus* (DeBiasse, Richards, & Shivji, 2010; Lacson et al., 1989) . This is likely because *P. marginemaculatus* is much more dispersal limited than a marine fish. Interestingly, the least divergent locality pairs of theses marine species included Key Largo, which is the same pattern we find in our study. This matching pattern supports the idea that currents play a major role in *P. marginemaculatus* genetic structure.

Pairwise *G*_ST_ showed that the greatest divergence was between Key Largo and the other regions (Figure 2). This could be caused by migrants from the Bahamas, the closest islands of which are ca. 100 km from Key Largo. Surprisingly, the mainland had low *G*_st_ estimates with the Upper and Lower Keys. We posit that this is due to ongoing gene flow between these regions. Ocean currents likely push rafting *P. marginemaculatus* to the Lower Keys as might major weather events including hurricanes (Fleming & Murray, 2009). Experiments with GPS‐equipped buoys show that this is a major current pathway (Lee & Smith, 2002) and *P. marginemaculatus*, with the ability to breathe underwater, are aptly suited to survive the voyage (Hebets & Chapman, 2000). Other research has emphasized the importance of ocean currents in the Florida Keys in genetic structure, but this has been limited to marine species. For example, a study of three marine invertebrates showed high gene flow and connectivity across the Keys, with a pattern of southern migration (Richards et al., 2007). The salt marsh snake (*Nerodia clarkii*), which is somewhat restricted to shallow waters, showed genetic structure between Upper and Lower Keys that was explained by IBD (Jansen et al., 2007). In general, our SDM predicted habitat suitability for *P. marginemaculatus* in parts of Monroe, Miami‐Dade, Collier, Lee, and Hendry counties. This limited range generally matches museum records, with occasional records from counties as far North as Charlotte county (Quintero, 1981). These northerly records could be from sparse populations, where detecting the species is difficult, or collections could be made by vagrant alloanthropic individuals associated only with human structures, and not viable populations. Additionally, model predictions of habitat suitability within the urban areas of southeastern Florida mainland should be cautiously interpreted as realized viable habitat is much less because of human development.

Few studies of Florida biogeography have been conducted, but generally align with our results. Ant gut microbiota show similar genetic structure between the Upper and Lower Keys, but also shoe divergence among the Lower Keys, which might be indicative of Caribbean migration (Hu et al., 2014). The mosquito *A. aegypti* showed practically no genetic structure among the Florida Keys, likely because they disperse via flight (Brown et al., 2013). Shrestha et al. (2015) proposed that the *C. incanum* spread southwesterly to colonize new Keys as they formed, with Lower Key populations being the youngest. Lastly, ant gut microbiota showed genetic structure between the Upper and Lower Keys (Hu et al., 2013).

Our models identified areas of tropical hardwood hammocks and pine rocklands as the most suitable habitat types for *P. marginemaculatus*. Indeed, this is where almost all of our observations occurred and is corroborated by published collection sites of the species for laboratory research (Hebets & Chapman, 2000; Weygoldt, 2000). The two habitat types, being at relatively high elevation, are the primary targets for human development in Florida (Noss, LaRoe, & Scott, 1995; Snyder et al., 1990). Thus, our modeling results show that *P. marginemaculatus* suitable habitats are also areas where human disturbance has been, and continues to be, an imminent threat to the habitat and species.

Pine rockland forests, once common throughout southeastern Florida, are now one of the most threatened habitats globally, with at least 98% global loss (Noss et al., 1995). This includes ca. 8,000 ha in Everglades National Park and a mere 920 ha outside the park's boundary (Bradley, 2005). Pine rockland habitat was identified as one of the most suitable habitats based not only on our models that included land use categories but also those with only climate variables and geology. Pine rockland habitats occur on exposed limestone substrates where limestone rock outcroppings are common and provide important microhabitat for *P. marginemaculatus* (Chapin & Hebets, 2016; Hebets & Chapman, 2000). Human development, fire suppression, and climate change have altered or entirely removed many areas once dominated by this community (Kautz & Cox, 2001; Possley, Woodmansee, & Maschinski, 2008; Ross et al., 1994). This habitat loss has resulted in five federally listed animal and 21 rare, endemic plant species sympatric with *P. marginemaculatus,* all of which are dependent on remaining fragments of pine rockland habitat (Florida Natural Areas Inventory, 2010). Furthermore, limestone rocks that make up critical habitat for *P. longipes* are often collected and used for construction and landscaping.

Some areas of pine rockland and upland hardwood forest are protected today. These include patches within Everglades National Park, Big Cypress National Preserve, Key Deer National Wildlife Refuge, and state‐managed lands. Most habitat outside of these protected areas have already been destroyed, and many surviving fragments remain threatened and at risk of extirpation within the areas of Miami, surrounding suburban areas, and in the tourist‐dominated Florida Keys. This has dramatic implications for *P. marginemaculatus* and the endemic, endangered community in which they occur.

Human development is not the only threat to pine rockland and upland hardwood forest habitats; sea level rise brought on by human‐induced climate changes is also threatening these habitats (Maschinski et al., 2011; Ross et al., 2009). In this sense, increases in the frequency and intensity of hurricane storm surges reshape pine rockland and similar vegetation communities (Ross et al., 1994). It remains unknown how climate‐induced changes in storm systems may have already impacted the considerably fragile extant *P. marginemaculatus* populations. We can, however, glean insight from studies of other species. For example, Hurricane Andrew dramatically altered pine rockland communities when it struck portions of Everglades National Park and Big Cypress National Preserve, leading to ca. 90% mortality of mature pine trees (Maguire, 1995) which negatively impacted plant and animal communities (Lloyd & Slater, 2012; Orr & Ogden, 1992; Williams, Wang, Borchetta, & Gaines, 2007). Major storms and human disturbance not only alter habitats but can also lead to isolation of populations and reshape population genetics of species at risk (e.g., Villanova, Hughes, & Hoffman, 2017). Our research was on specimens collected in 2015, prior to the 2016 Hurricane Matthew and 2017 Hurricane Irma events. Future research will benefit from examining the impacts of these and other storms on population structure of *P. marginemaculatus* in southern Florida.

Both our genetic and ecological results are limited by our dataset, which is constrained in both time and space. Spatially, we only sampled *P. marginemaculatus* in southern Florida and the Florida Keys archipelago, but the species occurs as far southwest as Hispaniola, including populations in the Bahamas, Cuba, Jamaica, and the Turks and Caicos (Quintero, 1981). In particular, gene flow from the Bahamas and Cuba to Key Largo and the Lower Keys could be ongoing, but this remains unexamined. Temporally, we excluded historical samples with the concern that they would not inform modern biogeographic patterns. While historical material would increase sample sizes, our modern collections span the spatial range of Florida historical samples bar northern limits, where populations may no longer occur. Sampling was uneven across sites, which could bias results. Lastly, we used only one sequence in our analysis. We chose a mtDNA marker because nDNA appears highly conserved in Amblypygi (Esposito et al., 2015), and mtDNA is thus, more informative. Caution should be taken, however, in interpreting results from only one mtDNA marker, and future research using more thorough genomic sequencing could reveal more high‐resolution biogeographic patterns.

### 4.1 Conservation

Our results point to a primary threat to the population health of *P. marginemaculatus* in the wild: habitat loss by human development. Approximately 77,000 people permanently reside in the Florida Keys (US Census Bureau). Given the small land area of the archipelago, this accounts for an average density of 205.54 people per square kilometer and leaves approximately 150 km^2^ uninhabited by humans, much of which is seasonally or permanently flooded habitat unsuitable for many terrestrial species. Furthermore, this does not include the impact from commercial, transportation, and utilities developments. Fortunately, much of the remaining habitat for *P. marginemaculatus* is within protected areas managed by federal and state agencies. This, however, does not protect habitats from all threats, including the impacts of increased hurricanes, sea‐level rise, poaching, and microhabitat alterations.

Secondarily, *P. marginemaculatus* is collected from the wild for sale in the pet trade. While we do not have data on the number of individuals collected for sale in the pet industry, personal observations lead us to believe that it must be in the hundreds to thousands. Wild populations of *P. marginemaculatus* would benefit from captive breeding that allows these fascinating animals to be kept as pets without reducing numbers in the wild. More research on wild populations is needed to assess population health, and we encourage researchers to conduct both field and laboratory studies on these fascinating organisms.

## CONFLICT OF INTEREST

None declared.

## AUTHOR CONTRIBUTIONS

K.J.C. conceived the study; K.J.C and D.E.W collected and extracted samples; I.A. and P.W. sequenced, cleaned, and aligned DNA; K.J.C. analyzed genetic data; D.E.W. analyzed ecological data; K.J.C. and D.E.W. drafted the manuscript; All authors contributed to writing and editing.

## DATA ACCESSIBILITY

DNA sequences have been under accession number MH478912‐MH479012.
